# Supplementary material for: The Advantage of Using an Optical See-Through Head-Mounted Display in Ultrasonography-Guided Needle Biopsy Procedures: A Prospective Randomized Study
Source: J Clin Med. 2023 Jan 8;12(2):512. doi: 10.3390/jcm12020512 (PMC9865023; doi:10.3390/jcm12020512)
Supplement: Supplementary file 1 [file jcm-12-00512-s001.zip › jcm-2031737-supplementary.pdf]

Please mark the point that represents your stress on each line. Signature ______________

(1) Level of stress while you identified the image of target lesion on the monitor

a. While using the standard ultrasound display

0 100

b. While using the optical see-through head-mounted display

0 100

(2) Level of stress while you inserted the needle into the breast phantom

a. While using the standard ultrasound display

0 100

b. While using the optical see-through head-mounted display

0 100

(3) Level of stress while you advanced the needle into the phantom

a. While using the standard ultrasound display

0 100

b. While using the optical see-through head-mounted display

0 100

(4) Level of stress while you punctured the target lesion

a. While using the standard ultrasound display

0 100

b. While using the optical see-through head-mounted display

0 100

Supplementary Figure S1

The questionnaire that participants were asked to fill out. The participants placed a mark at a point on each line to reflect their stress level at each step of the procedure. The scale ranged from 0 (left, least extreme) to 100 (right, most extreme). The visual analog scale (VAS) score was determined by measuring in mm from the left end of the line to the point marked by the participant.
